# Minimally Invasive Image-Guided Gut Transport Function Measurement Probe

**DOI:** 10.3389/fphy.2021.735645

**Published:** 2021-09-20

**Authors:** David O. Otuya, Evangelia Gavgiotaki, Camella J. Carlson, Serena Q. Shi, Ariel J. Lee, Alexander A. Krall, Anita Chung, Catriona G. Grant, Nitasha M. Bhat, Peter Choy, Sarah L. Giddings, Joseph A. Gardecki, Jay R. Thiagarajah, Steven M. Rowe, Guillermo J. Tearney

**Affiliations:** 1 Wellman Center for Photomedicine, Massachusetts General Hospital, Boston, MA, United States; 2 Harvard Medical School, Boston, MA, United States; 3 University of Pennsylvania, Philadelphia, MA, United States; 4 Korea Advanced Institute of Science and Technology, Daejeon, South Korea; 5 Division of Gastroenterology, Hepatology and Nutrition, Boston Children’s Hospital, Boston, MA, United States; 6 Department of Medicine, University of Alabama at Birmingham, Birmingham, AL, United States; 7 Gregory Fleming James Cystic Fibrosis Research Center, Birmingham, AL, United States; 8 Department of Pathology, Massachusetts General Hospital, Boston, MA, United States; 9 Harvard-MIT Division of Health Sciences and Technology (HST), Boston, MA, United States

**Keywords:** intestinal permeability, gut potential difference, transnasal probe, m-mode OCT imaging, duodenum diagnosis

## Abstract

**Introduction:**

Diseases such as celiac disease, environmental enteric dysfunction, infectious gastroenteritis, type II diabetes and inflammatory bowel disease are associated with increased gut permeability. Dual sugar absorption tests, such as the lactulose to rhamnose ratio (L:R) test, are the current standard for measuring gut permeability. Although easy to administer in adults, the L:R test has a number of drawbacks. These include an inability to assess for spatial heterogeneity in gut permeability that may distinguish different disease severity or pathology, additional sample collection for immunoassays, and challenges in carrying out the test in certain populations such as infants and small children. Here, we demonstrate a minimally invasive probe for real-time localized gut permeability evaluation through gut potential difference (GPD) measurement.

**Materials and Methods:**

The probe has an outer diameter of 1.2 mm diameter and can be deployed in the gut of unsedated subjects via a transnasal introduction tube (TNIT) that is akin to an intestinal feeding tube. The GPD probe consists of an Ag/AgCl electrode, an optical probe and a perfusion channel all housed within a transparent sheath. Lactated Ringer’s (LR) solution is pumped through the perfusion channel to provide ionic contact between the electrodes and the gut lining. The optical probe captures non-scanning (M-mode) OCT images to confirm electrode contact with the gut lining. A separate skin patch probe is placed over an abraded skin area to provide reference for the GPD measurements. Swine studies were conducted to validate the GPD probe. GPD in the duodenum was modulated by perfusing 45 ml of 45 mM glucose.

**Results:**

GPD values of −13.1 ± 2.8 mV were measured in the duodenum across four swine studies. The change in GPD in the duodenum with the addition of glucose was −10.5 ± 2.4 mV (*p* < 0.001). M-mode OCT images provided electrode-tissue contact information, which was vital in ascertaining the probe’s proximity to the gut mucosa.

**Conclusion:**

We developed and demonstrated a minimally invasive method for investigating gastrointestinal permeability consisting of an image guided GPD probe that can be used in unsedated subjects.

## INTRODUCTION

The intestinal epithelium provides a barrier against antigens and toxins from the environment and selectively allows absorption of essential nutrients, electrolytes, and water into the body. Normal intestinal permeability (IP) refers to the selective movement of ion, nutrients and water across the mucosa from the intestinal lumen into the circulating blood [1]. Several studies have shown that a breakdown of the epithelial barrier and subsequent alteration of intestinal permeability is associated with inflammation, infection and autoimmune diseases [2–4].

Changes in intestinal permeability have been specifically linked to a wide variety of diseases and syndromes including celiac disease [5], type II diabetes (T2D) [5–8]; inflammatory bowel disease (IBD) [9–11], irritable bowel syndrome [12,13], environmental enteric dysfunction [14,15], obesity [16,17], chronic fatigue [18], fibromyalgia [19] and colon cancer[20,21].

The standard method for measuring intestinal permeability in human subjects involves the administration of oligosaccharides of different molecular weights [22,23]. Non-metabolized pairs of sugars such as lactulose, mannitol or rhamnose are orally administered and urine elimination measured over a 3–24 h period. Although, the assessment of intestinal permeability by dual sugar absorption has been proven to be non-invasive and safe, it is time consuming since urine samples are collected between 3–24 h after sugar-probe administration and then shipped to a centralized facility for analysis, taking days to weeks [24,25]. Interpretation of the data can be confounded by a number of factors including, low urinary volumes, variable gut surface area and intestinal transit times. In addition, quantitative analysis of the urine sample requires high pressure liquid chromatography (HPLC) in combination with mass spectroscopy or direct enzyme-linked immunosorbent assay (ELISA) [25]. Lastly, the ability to collect clean urine samples without fecal contamination in certain populations such as infants, children and the elderly can be challenging, affecting the utility of the dual-sugar test for intestinal permeability evaluation in these population groups [14].

Other methods including assaying endogenous intestinal biomarkers in the serum, such as zonulin (ZO), intestinal fatty-acid binding proteins (FABPs), and tight junction (TJ) proteins, have been proposed for assessment of altered barrier integrity [26]. Bacteria or bacterial products have also been used to indicate changes of barrier integrity [27]. While simpler and less time consuming than the dual sugar method, it is currently not clear whether there is a sufficient correlation between these biomarkers and functional gut permeability *in vivo* [25,28].

The Ussing chamber, developed for the measurement of electrolyte and nutrient transport across epithelial tissues, offers the ability to measure permeability and ion transport in specific regions of the GI tract. However, this method measure tissues in *ex vivo* conditions requiring invasive collection via an intestinal biopsy sample, with subsequent issues related to tissue viability during measurements [25]. Since this method of gut permeability assessment uses small biopsies from specific areas of the gut, it is challenging to measure gut permeability over wide areas using the Ussing chamber.

There are numerous studies that have shown the applicability of potential difference (PD) measurements to record intestinal permeability alterations [29–34]. These experiments used probes made of salt bridges that consisted of 3% agar in saturated KCl, either in isolation or in combination with a tube for constant Ringer’s solution perfusion, to provide ionic contact between the electrodes placed outside the body and the mucosal lining of the gut. These probes were placed in the gut invasively through surgical access or *trans*-orally under fluoroscopic guidance. The methods reported above used varying unrefined probes, that were not commercially available or standardized and were prone to errors due to the inability to ascertain probe-mucosal contact when measuring PD.

In this paper, we propose a gut PD measurement (GPD) probe with a capability for real-time measurement of gut potential difference, providing dynamic real-time measurements of gut permeability. We have developed an image guided GPD measurement device that obviates the need for fluoroscopic guidance and consequent radiation exposure. The probe is a small caliber device that can be deployed via a *trans*-nasal guidance tube or endoscope. In addition, we have eliminated the need for an agar bridge, making the GPD probe a sterilizable, transportable, reusable, and convenient to use at the bedside in humans. We present the design of the GPD probe and show results to validate its safety and efficacy in measuring gut PD.

## MATERIALS AND METHODS

### *Trans*-Nasal GPD Probe

**FIGURE 1 |:**
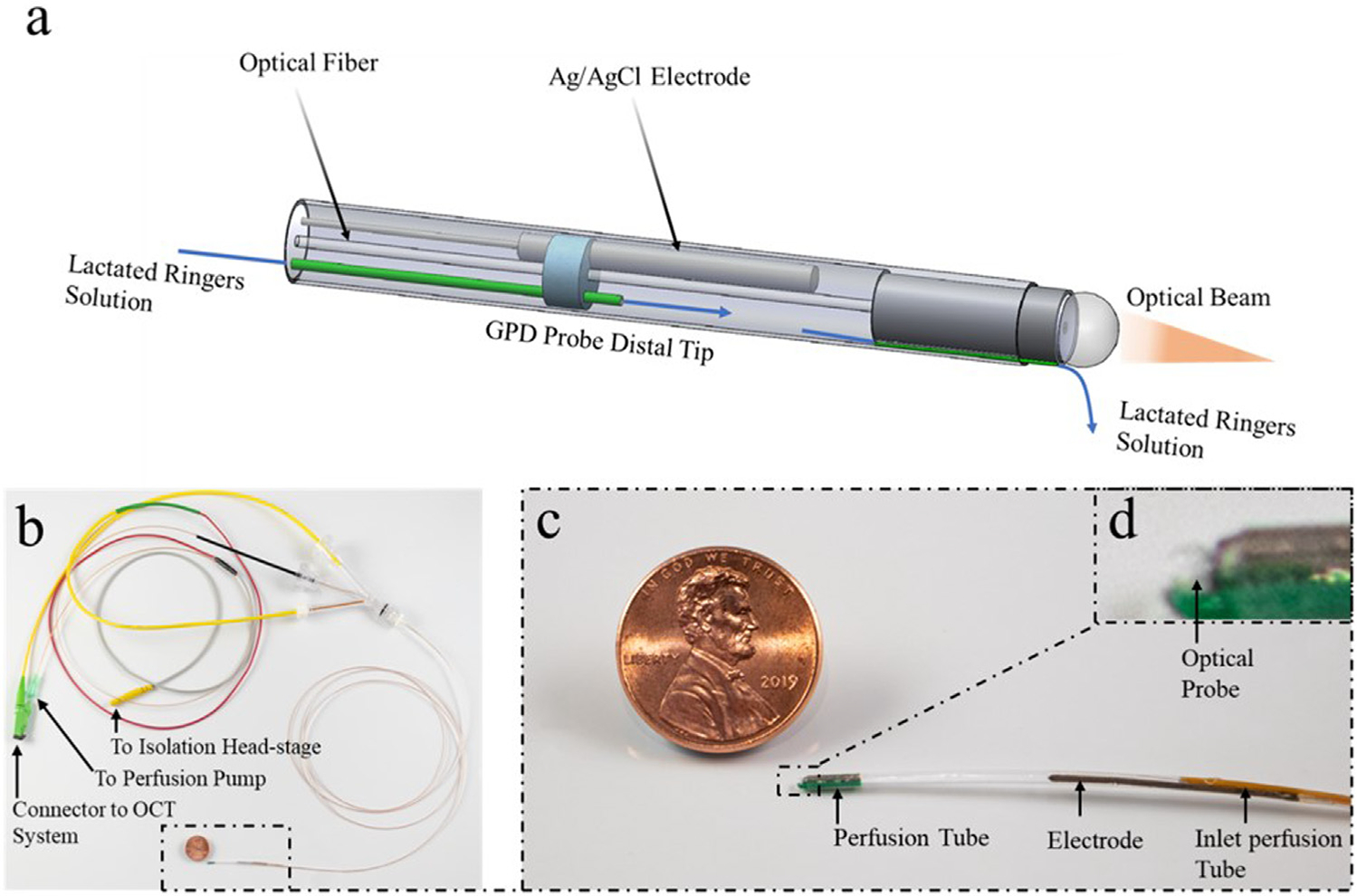
The GPD probe consists of a mini cell at the tip and the proximal connectors. **(A)** The tip is enclosed in a 1.2 mm outer diameter (OD) polytetrafluoroethylene (PTFE) tube with a 0.5 mm diameter Ag/AgCl electrode, an optical probe consisting of a ball lens and a single mode fiber (SMF), a perfusion tube that delivers lactated Ringer’s solution to the mini cell and perfusion channel that conducts a drip of lactated Ringer’s solution to the exteriorly located tissue. **(B)** The proximal end consists of a Luer lock connector to the perfusion tube from a perfusion pump, an optical connector from an OCT system to the optical probe and an electrical connector that links a wire from the Ag/AgCl to the isolation head-stage. **(C, D)** A Magnified view of the GPD probe.

A schematic of the GPD catheter is shown in Figure 1. The GPD probe used in this study was a 1.2-mm-diameter device that can be introduced into the GI tract transnasally via an introduction tube. The probe was enclosed in 1.2 m long, 1.2 mm outer diameter (OD) and 1.0 mm internal diameter (ID) polytetrafluoroethylene (PTFE) tube (ZEUS^®^, SC, United States). The device consisted of a 0.5 mm OD Ag/AgCl electrode (World Precision Instruments (WPI), FL, United States ) contained in a mini cell located at the distal end of the probe. A PTFE/composite perfusion tube of OD 0.47mm and 0.26 mm ID (Microlumen^®^, FL, United States ) was placed inside the entire length of the probe to transport Ringer’s solution from a perfusion pump outside the body to the mini cell at the distal end of the probe. Ringer’s solution was injected via the perfusion port to provide ionic contact between the electrode and the intestinal mucosa. A single mode optical fiber (SMF) placed in the probe carried light to obtain non-scanning (M-mode) optical coherence tomography (OCT) images at the probe’s tip to determine when it is in contact with mucosa and to provide tissue morphological information. A reference probe was inserted subcutaneously or implemented via a 3 M^™^ Red Dot^™^ ECG monitoring electrode skin patch, attached over an abraded area of the skin. The GPD was defined as the voltage measured across the *trans*-nasal GPD probe and the reference electrode.

In humans, the concept will involve inserting the probe into the gut using either OCT image guidance or an equivalent method to confirm anatomical placement [35,36]. When using OCT for placement, a separate introduction tube containing a mechanically scanning optical probe in its working channel will be deployed in the gut. The B-mode frames provided by the scanning optics will be used to localize the introduction tube in the gut. After retraction of the optics, the GPD probe will be inserted through the working channel of the introduction tube. After the probe is situated in the duodenum, fluid will be perfused via the port and the probe brought in contact with the mucosa as confirmed with OCT. GPD will then be measured.

### GPD Measurement System

**FIGURE 2 |:**
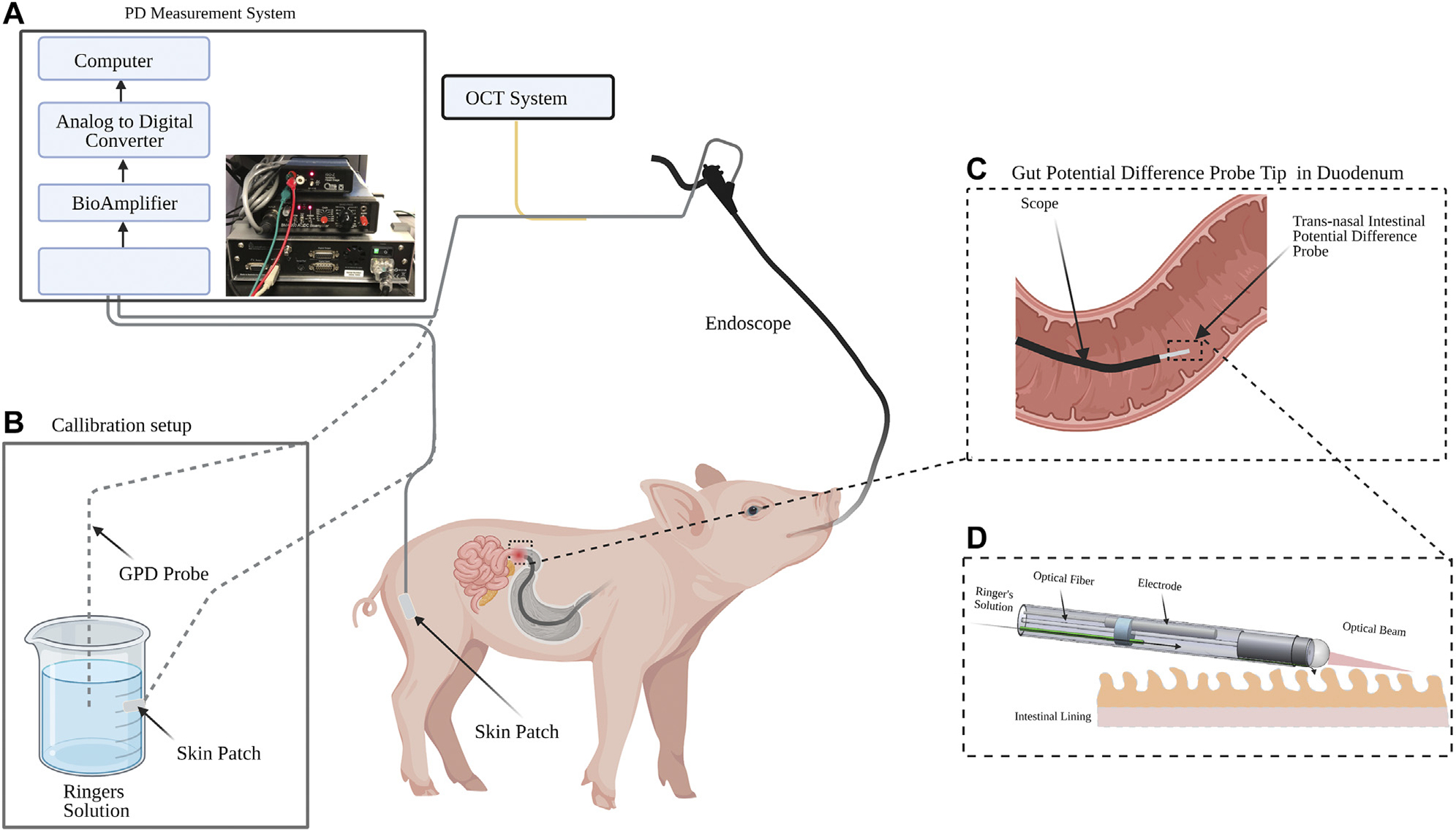
Experiment schematic for gut potential difference (GPD) measurement in swine. **(A)** PD measurement system consisting of an isolation head-stage, a bioamplifier, an analog-to-digital converter and a computer. **(B)** An offset zeroing setup made of a cup filled with Ringer’s solution and a skin patch. **(C)** The pig’s small intestine was intubated using a pediatric gastroscope and the IPD probe inserted through the working channel of the endoscope. The GPD signal was acquired via an isolation headstage and a bioamplfier in the GPD measurement system. The GPD signal was converted to a digital signal and logged using a computer. Optical coherence tomography (OCT) M-mode images were simultaneously acquired. **(D)** Detailed view of the duodenum and the GPD probe.

Figure 2A shows the GPD measurement system, which consisted of an isolation head-stage (ISO-Z, CWe Inc., PA, United States ) to which the *trans*-nasal GPD and reference probes were connected. The signal from the isolation head-stage was then connected to a bioamplifier (BMA-200, CWe Inc. PA, United States). The amplified signal from the bioamplifier was sent to a digitizer (Power Lab 4/26, AD instruments Inc., CO, United States) for conversion to a digital signal and logged with LabChart software (AD instruments Inc., CO, United States ) on a computer at a rate of 1 k sample/s. The GPD system was adapted from a nasal potential difference measurement system with similar equipment [37,38].

### Swine Studies

The GPD probe was connected to an OCT system consisting of an Axsun OCT engine (Excelitas, MA, United States ) operating at 1,310 nm center wavelength and acquiring OCT image frames at 100 kHz [39]. The optical probe acquired a non-scanning form of OCT images known as M-mode OCT. M-mode OCT was used to ensure that the probe was in contact with the mucosal tissue while the GPD values were measured. To validate the ability of the probe to measure real-time changes in GPD, a transient gut sodium ion concentration modulator (45 mM glucose) was perfused while potential difference measurements were taken. Electrogenic absorption of glucose causes a local reduction in the sodium ion concentration on the luminal side of the mucosa resulting in a change in the apical GPD. The swine studies were approved by the Massachusetts General Hospital (MGH) Institutional Animal Care and Use Committee (IACUC) protocol number 2016N000215.

### Procedure

1. Prior to the procedure, the swine were anesthetized and intubated by the staff at the Knight Surgery and Research Laboratory (KSRL) at MGH. Vitals were monitored to ensure the animal was in good physiological condition.
2. A convenient portion of the skin was shaved and abraded until interstitial fluid was exposed.
3. A skin patch was placed over the abraded area.
4. An endoscope was introduced *trans*-orally and advanced to the first segment of the duodenum.
5. Before any measurement was taken, the potential difference offset between the GPD probe and the skin patch was zeroed using the calibration setup shown in Figure 2B.
6. The reference cable from the isolation head-stage was then connected to the skin patch on the swine skin.
7. The GPD probe connected to the perfusion pump delivering Lactated Ringer’s solution to the probe at 1 ml/hr was inserted via the working channel of the scope.
8. Baseline GPD measurements were taken after 30 min of probe equilibration.
9. A secondary tube was inserted via the working channel in which glucose was perfused.

## RESULTS

**FIGURE 3 |:**
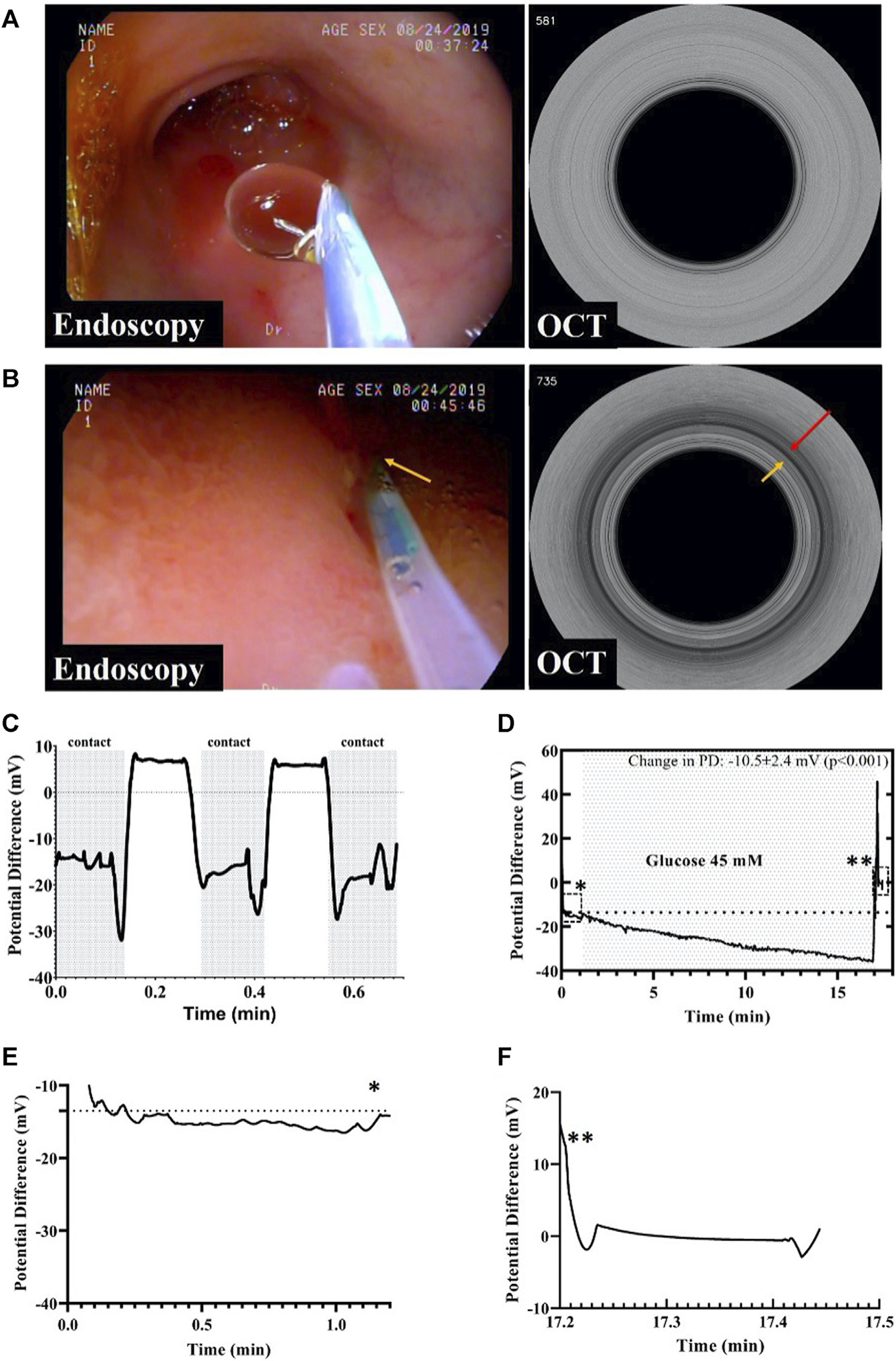
**(A)** Endoscopic image of the duodenum showing the GPD probe not in contact with the mucosa and the corresponding M-mode OCT image. **(B)** Endoscopic image showing the IPD probe in contact with the mucosa. An M-mode OCT frame corresponding to the endoscopy frame depicting the GPD probe in contact with the duodenal mucosa, as shown by the dark ring pointed by the red arrow. **(C)** GPD values measured by the probe in contact and not in contact with the mucosa. **(D)** This figure shows a change in the gut potential difference after glucose perfusion. The baseline GPD value changed in presence of 45 mM glucose solution by as much as −10.5 mV. The single asterisk demarcates the region enclosed by the dashed rectangle before glucose infusion while the double asterisks point to the region in the rectangle depicting the potential difference across the probe and the reference skin patch after the probe was retracted from the body. **(E)** The graph shows baseline GPD value of the duodenum before infusion of glucose. **(F)** After probe retraction from the duodenum, the graph shows the GPD between the GPD probe and the skin patch was about zero, indicating that the change in GPD prior to retraction was solely due to the presence of glucose, and not because of a walk-off between the probe and the reference skin patch. The asterisks indicate the positions of the graphs in **(E)** and **(F)** in the original graph in **(D)**.

Initially, an endoscope was safely deployed into the duodenum and through its working channel the GPD device inserted as shown in Figure 3A. Then, M-mode OCT images were acquired to ensure contact of the probe with the mucosa while PD values were recorded. The right half of Figure 3B demonstrates a representative M-mode OCT image with the red arrow indicating probe-tissue contact shown by the ring-like scattering pattern from the tissue that abuts the outside of the probe (yellow arrow). The measured average baseline GPD across four swine was −13.1 ± 2.8 mV.

Figure 3C shows a rapid time response of under 2 s when the GPD probe was not in contact with the mucosa was brought in contact with the tissue. We also evaluated the potential difference change that resulted from perfusion of 45 mM glucose over a duration of 60 s at a rate of 1 ml/s to stimulate gut electrolyte absorption as shown in Figure 3D. The total change in GPD was −10.5 ± 2.4 mV (*p* < 0.001), due to the glucose mediated active Na ^+^ transport across the mucosa. Figure 3E shows the initial baseline PD, while Figure 3F shows the PD between the probe and the reference skin patch after the device was retracted from the scope demonstrating the absence of a residual offset potential.

**FIGURE 4 |:**
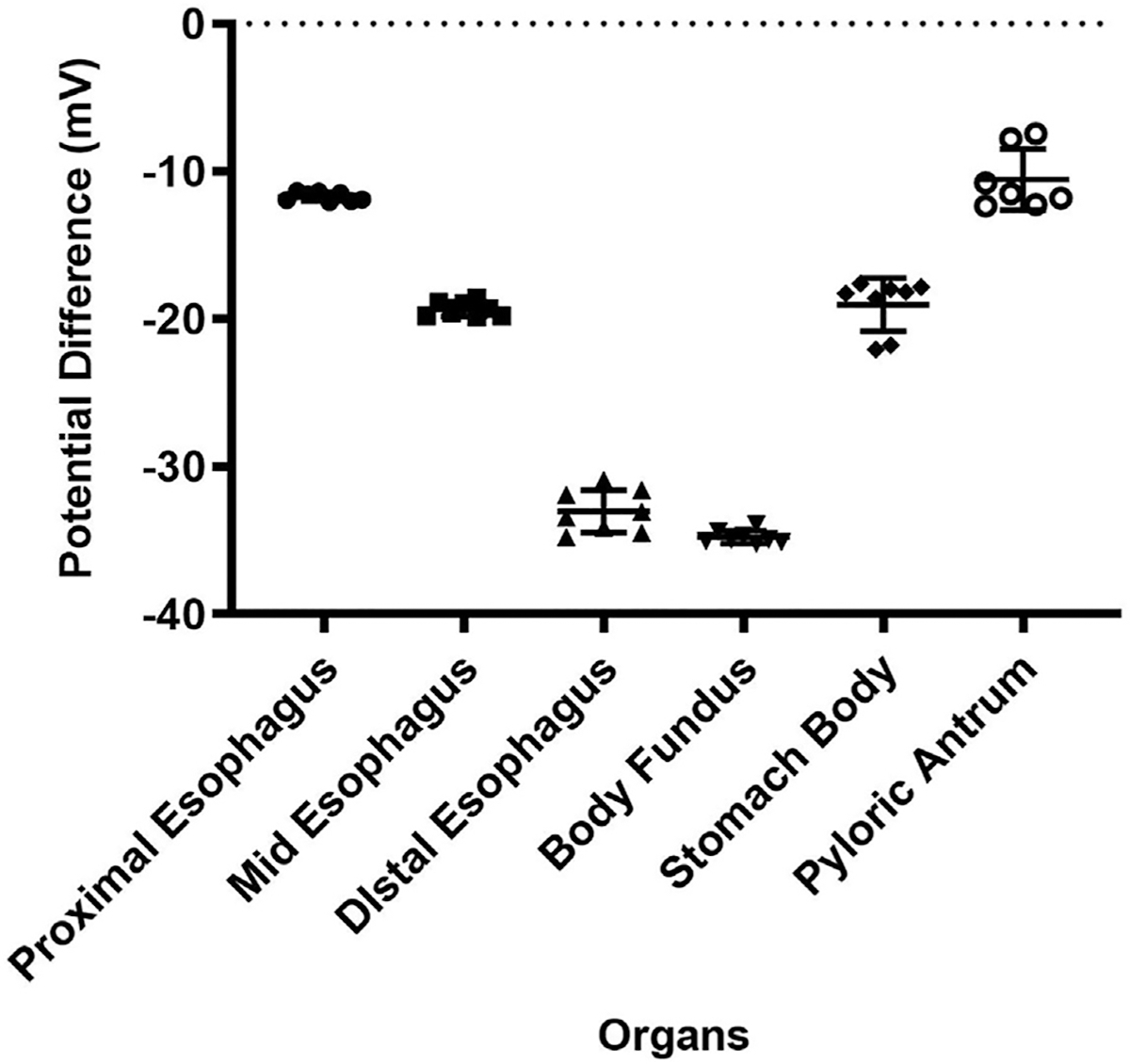
PD values of proximal esophagus, mid esophagus, distal esophagus, body fundus, stomach (body) and the pyloric antrum. Error bars indicate SD.

In addition, GPD measurements were acquired in different anatomical regions of the upper GI tract. Figure 4 demonstrates the PD values measured in three sections of the esophagus (proximal esophagus, mid esophagus and distal esophagus) measured over 15 s. The mean PD values measured in these organs was −11.6 ± 0.7, −20.4 ± 2.1, and −34.1 ± 1.3 mV for the proximal esophagus, mid esophagus and distal esophagus, respectively. In the stomach, as shown in the figure, the average PD measured in the stomach fundus, body and pyloric antral regions over a duration of 15 s was −34.2 ± 1.4, −20.2 ± 2.2, −12.7 ± 2.1 mV, respectively.

## DISCUSSION

Here, we have presented a design for a *trans*-nasal minimally invasive GPD measurement device. We validated the GPD probe in swine by measuring swine esophageal, stomach and duodenal potential difference. These duodenal potential difference values measured by the GPD probe approximated the PD values measured in human duodenum reported by Gustke et al [40]. Gastric PD measurements obtained with our GPD probe are different from those reported by Geall et al [31]. by about −10 to −15 mV. This discrepancy may be attributed to the lateral recumbent position of the swine during scoping that allowed for a small pool of gastric contents to remain lodged in the distal portion of the esophagus providing ionic connectivity to the stomach mucosa. The PD value measured with this probe in the proximal region of the esophagus is consistent with the potential difference values measured in the same region in humans [41]. Since there is insufficient information on GPD values in swine, further studies need to be done to establish the similarities and differences between human and swine GPD.

In addition to baseline GPD we also showed a change in duodenal PD after infusion of glucose solution and subsequent induction of electrogenic glucose uptake. This glucose absorption is coupled with sodium ion intake by the sodium/glucose cotransporter 1 (SLGT1) across the intestinal epithelium. Our results showed the expected decrease in transepithelial potential consistent with electrogenic Na^+^-glucose co-transport across the epithelium.

A major advantage of our device that enables blind (non-endoscopic) insertion is the integrated M-mode OCT imaging technology. M-mode imaging was able to indicate when the GPD probe was in contact with the intestinal wall. Thus, M-mode OCT allows for accurate GPD measurements by ensuring that PD values are acquired only when the device in contact with the mucosa. This feature is especially critical when the device is deployed *trans*-nasally, without endoscopic guidance, allowing for precise placement of the probe and accurate measurement of GPD. This was particularly important for measurement of positive potential changes that occur during intestinal electrolyte and fluid secretion, which would otherwise be erroneously attributed if the probe was not in contact with the mucosa. This will be particularly important in future studies caried out without the benefit of endoscopic guidance.

Real-time GPD measurement offers an attractive prospect for the investigation of gut transport function in a rapid and minimally invasive manner. As an example, targeted functional evaluation of ion channels such as the cystic fibrosis transmembrane conductance regulator (CFTR) can be performed with the aid of this GPD device. PD changes due to chloride transport via CFTR in response to electrochemical gradients using chloride free solution infusions with or without solutions containing cyclic adenosine monophosphate (cAMP) agonists, can be measured to ascertain the functional integrity of this critical ion channel [38]. Gut PD measurement may also play a role in further understanding the dynamics of secretory diarrheas such as cholera in humans [42] or deliver functional assessments of electrolyte and nutrient transport in a variety of disease states in infants including congenital diarrheas, allergic enteropathies and environmental enteric dysfunction [43–45]. Further, gut permeability to larger molecules can be measured and localized using this device. In contrast to the dual sugar method where heterogeneity of gut permeability may be hard to address, GPD measurements can provide spatial and segmental gut permeability data that may allow dynamic mapping of permeability along the length of the intestine. Lastly, PD measurements as a means for assessing “leaky-gut” conditions, may also be appealing in low resource settings. GPD measurement offers a low-cost and more practical alternative to the dual-sugar test, which does not require advanced ELISA/chromatography analysis of collected samples.

Several challenges may still hamper gut transport evaluation using this method. One major challenge is the temperature drift between the reference and the measurement probe brought about by a change in fluid temperature as it is perfused through the perfusion channel to the gut mucosa. This drift in temperature causes a small drift in the measured PD values [46]. Also, formation of bubbles within the perfusion channel creates areas of discontinuity affecting the PD values registered, a well-known impediment to nasal PD performance [37]. To minimize the formation of gas bubbles, we degas Ringer’s solution before each GPD measurement procedure by sonication for 30 min and then placing the solution in a vacuum at a pressure of about −75 kPa for another 30 min. Another issue that may affect PD measurement *in vivo* is the PD fluctuation due to movement of either the probe or the subject resulting in motion artifacts on the PD signal. External potential sources of noise to the PD signal that should be considered include background radiofrequency from the environment which can be eliminated by applying a low-pass filter to PD signal with cut-off frequency about 10 Hz, as has been successful employed in NPD [37].

We also note that GPD is an indirect measurement of gut permeability. While it is true that when the gut epithelium is completely permeable, GPD will go to zero, there are other processes that could cause the PD to diminish. For example, the efflux of negative ions from the lumen or influx of positive ions to the lumen will push PD values towards zero. For this reason, in the future, it will be important to measure both transepithelial voltage and current to determine the transepithelial resistance and short-circuit current [47]. While studies have shown a correlation between dysregulation of the tight junction proteins involved in gut permeability and a change in baseline GPD [48,49], more work needs to be done in the future to understand the correlation of GPD and ionic versus macromolecular intestinal permeability.

The minimally invasive gut transport measurement probe presented here is compatible with pediatric *trans*-nasal introduction in a similar manner to commonly used nasogastric/nasojejunal feeding tubes (7Fr). This device can be not only be used for assessment of gut permeability through PD measurement but could also be extended for evaluation of epithelial integrity and function in the airway and other luminal organs. This GPD device is also small enough to be deployed via the working channel of ultrasmall *trans*-nasal endoscopes, obviating the need for sedation that is routinely done during EGD and bronchoscopy. The ability to perform unsedated minimally invasive PD assessment opens opportunities for understanding both healthy and diseased tissues across populations, as well as allowing timely diagnoses of conditions in vulnerable groups such as infants, pregnant women, and the elderly.

## Data Availability

The raw data supporting the conclusions of this article will be made available by the authors, without undue reservation.
